# Human antigen R enhances the epithelial-mesenchymal transition via regulation of ZEB-1 in the human airway epithelium

**DOI:** 10.1186/s12931-018-0805-0

**Published:** 2018-06-04

**Authors:** Jian Sun, Xianmin Gu, Nan Wu, Pengju Zhang, Yi Liu, Shujuan Jiang

**Affiliations:** 1 Department of Respiratory Medicine, Shandong Provincial Hospital Affiliated to Shandong University, Jinan, 250021 People’s Republic of China; 2 Department of Biochemistry and Molecular Biology, Shandong University School of Medicine, Jinan, 250021 People’s Republic of China

**Keywords:** Chronic obstructive pulmonary disease, Cigarette smoke extract, Human antigen R, Epithelial–mesenchymal transition, Zinc finger E-box binding homeobox 1

## Abstract

**Background:**

Increasing evidence suggests that human antigen R (HuR) is involved in the epithelial-mesenchymal transition (EMT) process of several diseases. However, the role of HuR in EMT in the airway epithelial cells of patients with COPD remains unclear.

**Methods:**

BEAS-2B cells were cultured and treated with 3%CSE. Western blotting, RT-PCR and immunofluoresence were used to detect the expression of HuR, ZEB-1. RNAi was used to suppress HuR expression. Then knockdown of HuR, RT-PCR and Western blotting showed that with siHuR-1 and siHuR-3, clear suppression of HuR expression was confirmed. We chose siHuR-3, the most effective one, to proceed with subsequent experiments. Immunofluorescence analysis, western blotting were used to detect the expression of E-cadherin, vimentin, ZEB-1 and HuR.

**Results:**

We show that more HuR expression is enhanced in the airways epithelium of smokers with or without COPD than controls (nonsmoker non-COPD patients). However, there was no definite correlation between HuR expression and FEV1%. Further study reveals that knockdown of HuR significantly increases the apoptosis of BEAS-2B cells and down-regulates ZEB-1 expression.

**Conclusions:**

EMT is partially enhanced through the HuR-binding proteins and its post-transcriptional regulation role in airway epithelium in COPD.

## Background

COPD is a disorder with a high morbidity rate and is the third most common cause of mortality worldwide [1]. Chronic Obstructive Pulmonary Disease (COPD) is a common, preventable and treatable disease that is characterized by persistent respiratory symptoms and airflow limitation that is due to airway and/or alveolar abnormalities usually caused by significant exposure to noxious particles or gases. The prevalence of COPD is directly related to the prevalence of tobacco smoking. [2]. Because of the innumerable quantity of patients, the profound damage it causes and its burden on society, COPD has received considerable attention. The classic pathological changes of COPD are mucus hypersecretion, up-regulated inflammation and airway remodeling due to repeated damage and repair of the tissue [3]. Although many related studies have been conducted, there are still few treatments that can significantly decrease the mortality due to COPD [4].

Recently, several studies have indicated that EMT, a process by which epithelial cells acquire a mesenchymal-like cell phenotype, is closely related to the pathogenesis of COPD [5, 6]. Most of these studies investigated EMT-related molecules at the transcriptional and protein levels; however, to date, there have been few findings that were successfully applied to the clinic. We hypothesized that focusing on the post-transcriptional modification of mRNAs involved in EMT will be a good approach to overcome this obstacle.

HuR, a ubiquitously expressed RNA-binding protein (RBP), is one of the best-studied members of the post-transcriptional modification family [7]. HuR selectively binds to a large subset of mRNAs and influences the stability and/or translation of select mRNAs which are implicated in different pathologies, especially cancer and inflammation [8]. It has been reported that HuR mediates the EMT process in diabetic nephropathy [9]. However, the role of HuR in EMT in the airway epithelial cells of patients with COPD remains unclear. In the present study, we investigated whether HuR is involved in the cigarette smoke extract (CSE)-induced EMT process and its corresponding mechanism.

## Methods

### Patients

Lung tissues were obtained from 68 patients (18 non-smoking patients without COPD, 20 smokers without COPD, and 30 smokers with COPD) at Shandong Provincial Hospital (Jinan, China). A diagnosis of COPD was based on the GOLD guidelines [2]. No subjects received oral or inhaled corticosteroids before specimen collection. All the patients’ clinical data are shown in Table 1. Informed consent to undergo scientific research was obtained from all the patients before tissue collection, and the experiment was approved by the ethics committee at Shandong Provincial Hospital.

**Table 1:** Demographic characteristic of the subjects

	Non-smokers n = 18	Smokers n = 20	COPD^a^ n = 30
Sex (female/male)	13/5	1/19	3/27
Age (years)	52 ± 11	54 ± 10	58 ± 8^e^
Smoking history, pack-years^b^	–	26 ± 12	42 ± 25^e^
FEV1^c^, % predicted	99 ± 12	100 ± 11	66 ± 15^e^
FEV1/FVC^d^ %	85 ± 7	84 ± 7	57 ± 8^e^
GOLD stage			
1	–	–	6
2	–	–	19
3	–	–	5
4	–	–	–

^a^COPD, chronic obstructive pulmonary disease. ^b^Pack-year, 1 year smoking 20 cigarettes per day. ^c^FEV1, forced expiratory volume in 1 s. ^d^FVC, forced vital capacity. ^e^Values are given as mean ± s.d

### Immunohistochemistry

HuR and ZEB-1 were immunohistochemically assessed in formalin-fixed, paraffin-embedded lung tissues. Histological sections were sliced at a thickness of 4 μm and mounted on poly-L-lysine-coated slides. Immunohistochemical analysis was performed as previously described [10]. The primary antibodies targeted HuR (1:50) was purchased from Abcam and ZEB-1 (1:50) was purchased from Cell Signaling. Color development was performed using a DAB color devel-opment kit (ZhongShan Biotech). Images were captured using an OLYMPUS IX81 light microscope (Olympus, Tokyo, Japan) fitted with a SPOT camera. Image analysis was performed using mage-Pro Plus 6.0 software (Media Cybernetics, Silver Spring, MD, USA). All slides were analyzed in a single batch by a single experienced observer with quality assurance on randomly selected slides provided by a professional academic pathologist.

### Preparation of CSE

CSE preparation was based on the method previously described by Aoshiba et al. [11]. Briefly, one commercial cigarette (Hatamen), which contains 11 mg of tar and 0.8 mg of nicotine, was used in this study. A filter-free cigarette was combusted using a syringe-driven instrument, and the smoke was bubbled through 20 ml of serum-free RPMI 1640 culture medium. The resulting suspension was adjusted to a pH of 7.4 and filtered using a 0.22-μm pore filter. This solution was regarded as a 100% CSE solution and was used within 30 min after preparation.

### Cell culture

Human bronchial epithelial (BEAS-2B) cells were obtained from ATCC. Cells were routinely cultured in high-glucose RPMI 1640 medium (HyClone) supplemented with 10% fetal bovine serum (Biological Industries, Israel), 100 units/ml penicillin (Invitrogen) and 100 units/ml streptomycin (Invitrogen) and maintained at 37 °C in a 100% humidified atmosphere containing 5% CO_2_.

### Real-time PCR (RT-PCR)

Total RNA was extracted from BEAS-2B cells using Trizol reagent (Invitrogen) based on manufacturer’s protocol. The reverse transcription was performed according to the specification of RevertAid First Stand cDNA Synthesis Kit (Thermo Scientific). RT-PCR was conducted using SYBR Premix EX Taq (Takara), in a total reaction volume of 20 μl. The relative expression levels of target mRNAs were normalized to human β-actin expression. Primers sequences were shown as follows. HuR: forward, 5’GGCGAGCATACGACA3’, reverse, 5’TATTCGGGATAAAGTAGC3’; β-actin: forward, 5’ AGTTGCGTTACACCCTTTCTTG3’, reverse, 5’ CACCTTCACCGTTCCAGTTTT3’.

### Western blot analysis

Total cellular lysates were prepared as previously described [12]. Nuclear and cytoplasmic proteins were extracted using Nuclear and Cytoplasmic Protein Extraction Kit (Beyotime). 30 μg of each protein sample was fractionated in a 10% SDS–PAGE gels. The membranes were incubated with antibodies against HuR (1:5000, Abcam), E-cadherin (1:1000, Cell Signaling), vimentin (1:1000, Cell Signaling), ZEB-1 (1:500, Cell Signaling), HDAC1 (1:1000, Abcam) and β-actin (1:1000, ZSGB-BIO). HDAC1 was used as nuclear protein internal control, and β-actin was used as cytoplasmic and total protein internal controls.

### Small interfering RNA (siRNA) gene silencing

HuR siRNA (siHuR) and negative control siRNA (siNC) were purchased from RiboBio (Guangzhou, China). siRNA constructs were transfected using the riboFECT™ CP Reagent (RiboBio) according to the manufacturer’s instructions. The knockdown efficiency was tested at both mRNA and protein levels 48 h after transfection. At 12 h after transfection, the medium was changed, and further experiments were conducted. siRNA sequences were as follows: siHuR-1, forward: 5′ - GGAGAACGAAUUUGAUCGU dTdT - 3′, reverse: 3′ - dTdT CCUCUUGCUUAAACUAGCA - 5′; siHuR-2, forward: 5′ - GUCCUCGUGGAUCAGACUA dTdT - 3′, reverse: 3′ - dTdT CAGGAGCACCUAGUCUGAU - 5′; siHuR-3, forward: 5′ - GGUUGCGUUUAUCCGGUUU dTdT - 3′, reverse: 3′ - dTdT CCAACGCAAAUAGGCCAAA - 5′.

### Plasmids and transfection

Human ZEB-1 expression vector was purchased from Public Protein/Plasmid Library (Nanjing, China). X-tremeGENE HP DNA Transfection Reagent (Roche, Indianapolis, IN, USA) was used to transfect the plasmids into indicated cells. The transfection procedures followed the protocol of the manufacturer.

### Immunofluorescence

After the cells received their respective treatments, immunofluorescent staining was performed as previously described [13]. Samples were incubated with primary antibodies against HuR (1:100), vimentin (1:100) and E-cadherin (1:200) overnight followed by treatment with a secondary antibody labelled with Alexa Fluor 488 (Beyotime). Images of the cells were captured on an inverted fluorescence microscope.

### Statistical analysis

All experiments were repeated at least three times. SPSS 17.0 software (SPSS Inc.) was used for data statistical analysis. The Mann-Whitney test was applied for comparisons between the patient groups. The Spearman test was used for correlation analyses. Student’s t-test was applied to the in vitro experiments. *P* < 0.05 was considered statistically significant.

## Results

### HuR expression was increased in airway epithelium of COPD subjects

**Fig. 1:**
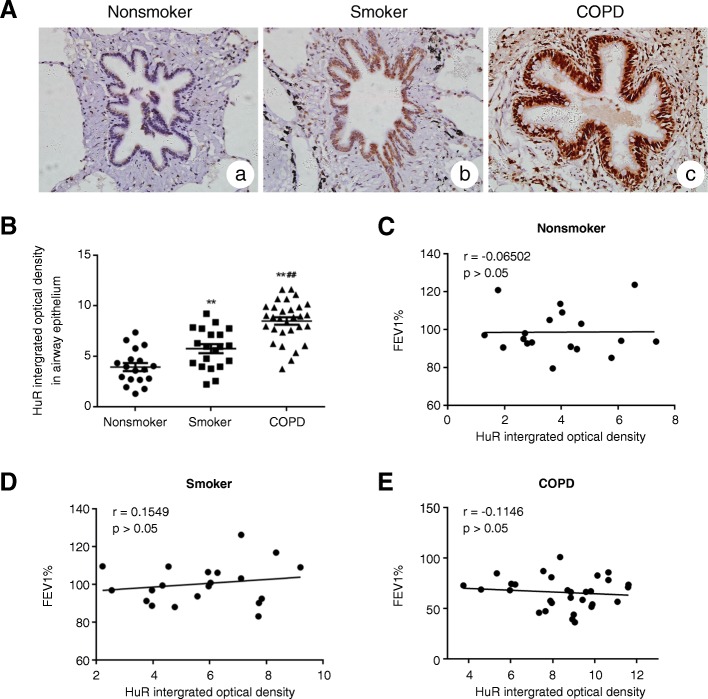
HuR expression and its correlation with parameters of lung function in the airway epithelium. Immunohistochemical assessment of HuR expression in non-smoking subjects (*n* = 18), smokers without COPD (*n* = 20), and smokers with COPD (*n* = 30). (**a**) Representative HuR immunostaining (brown staining) in the airways of a nonsmoker (*a*), a smoker without COPD (*b*), and a smoker with COPD (*c*). (**b**) Quantification of HuR protein levels in the airway epithelium using the integrated optical mean density. The expression of HuR was not correlated with the predicted FEV1% in the airway epithelium of nonsmokers (**c**), smokers without COPD (**d**), and smokers with COPD (E) by Spearman’s correlation test. The values are given as the mean ± s.e.m. **P < 0.01 compared with nonsmokers and ##*P* < 0.01 compared with smokers without COPD

To assess HuR expression in airway epithelium of COPD subjects, lung sections from non-smoking patients without COPD patients, smokers without COPD, and smokers with COPD were stained by immunohistochemistry techniques. As shown in Fig. 1A, more intense HuR staining was observed in the airway epithelia of smokers with or without COPD than controls (non-smoking patients without COPD), especially among smokers with COPD. Quantitative analysis of the HuR staining (Fig. 1B) showed that HuR expression was significantly increased in smokers with COPD than in smokers (*P* < 0.01) and nonsmokers (*P* < 0.01) without COPD. The HuR expression was also higher in the smokers without COPD than in nonsmokers (*P* < 0.01). Then the correlation between HuR expression and FEV1% was analyzed and the results showed that there was no significant correlation between FEV1% and HuR expression (Fig. 1C-E).

### CSE elevated HuR expression and activity in BEAS-2B cells

**Fig. 2:**
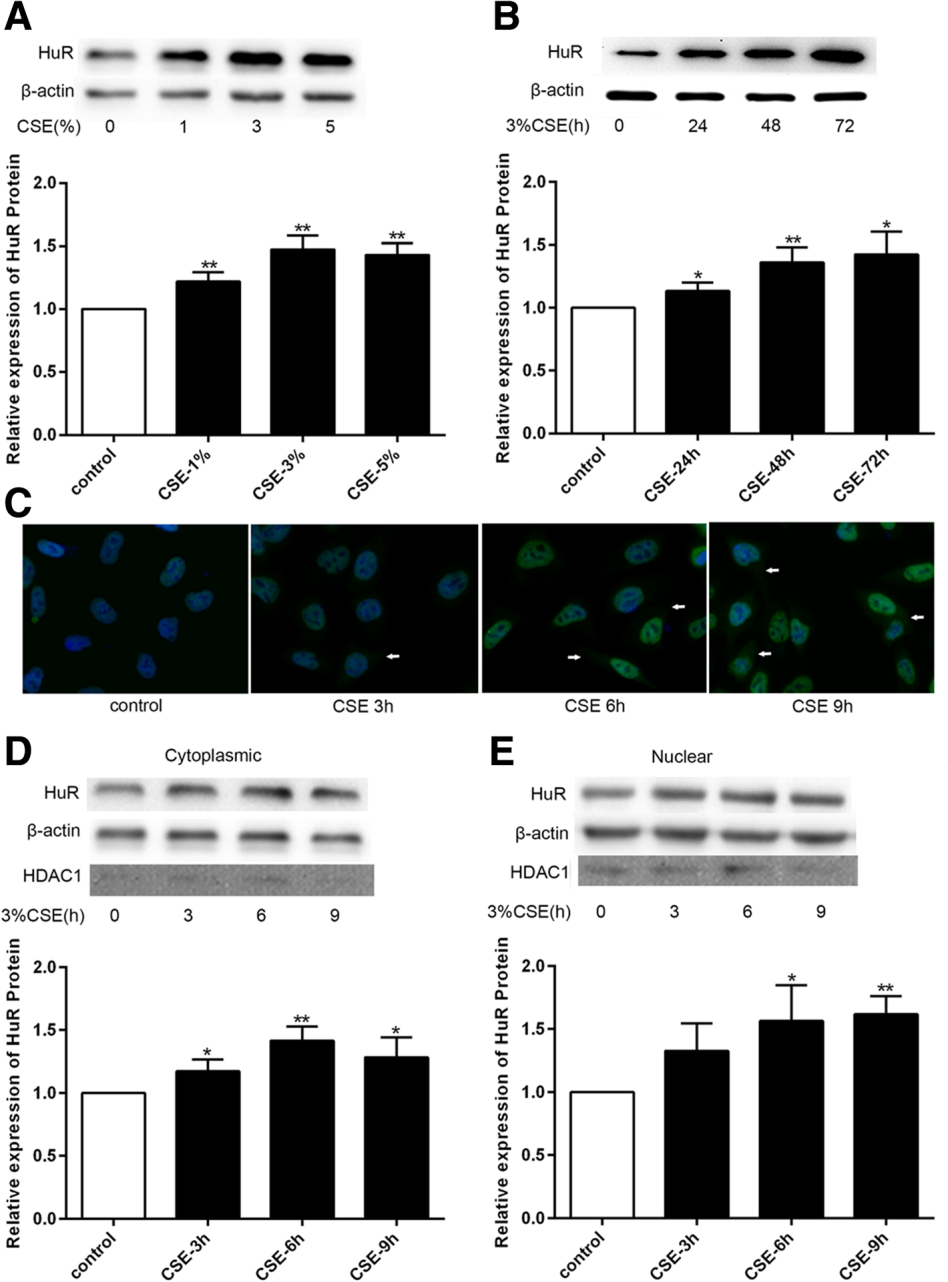
CSE increased HuR expression and enhanced the cytoplasmatic translocation of HuR in BEAS-2B cells. **a** HuR expression was examined by Western blot with various concentrations of CSE for 48 h. **b** HuR expression was examined by Western blot with 3% CSE for different time periods. **c** Immunofluorescence analysis of the subcellular distribution of HuR in BEAS-2B cells treated with 3% CSE for different time periods. The arrows refer to the cytoplasmic distribution of HuR. Western blot of HuR expression levels in the cytoplasm (**d**) and nucleus (**e**) in cells treated as described in (**c**) compared with the cytoplasmic marker β-actin and the nuclear marker HDAC1, respectively. Each value is presented as the mean ± SD from three independent experiments. *P < 0.05, **P < 0.01 compared with control group

Since cigarette smoking is the most common risk factor for COPD, BEAS-2B cells were treated with different concentrations of CSE for different time to model this microenvironment. Resultantly, compared with the control group (no CSE treatment), a significant elevation of HuR expression was observed after 48 h treatment of 1, 3 and 5% CSE (Fig. 2a). Of note, upon exposure to 3% CSE, the cells exhibited the most pronounced elevation of HuR expression. Furthermore, CSE was also shown to increase HuR expression in a time-dependent manner (Fig. 2b). These findings suggested that CSE stimulation could induce HuR expression.

Along with its expression levels, HuR function is also associated with its subcellular distribution. More proteins will be shuttled to the cytoplasm when HuR is appropriately activated [8, 14]. We next investigated the influence of CSE on the subcellular distribution of HuR. Immunofluorescence analysis illustrated that CSE stimulation for 3, 6 and 9 h significantly elevated the cytoplasmatic levels of HuR than those in control cells (Fig. 2c). Similar evidence was observed using Western blot analysis (Fig. 2d). At the same time, in the nuclear fraction, elevation of HuR expression was observed when the CSE treatment was prolonged from 3 h to 9 h (Fig. 2e). Taken together, besides enhancing HuR expression, CSE might promote HuR translocation from nucleus to cytoplasma as well.

### HuR was required for CSE-induced EMT in BEAS-2B cells

**Fig. 3:**
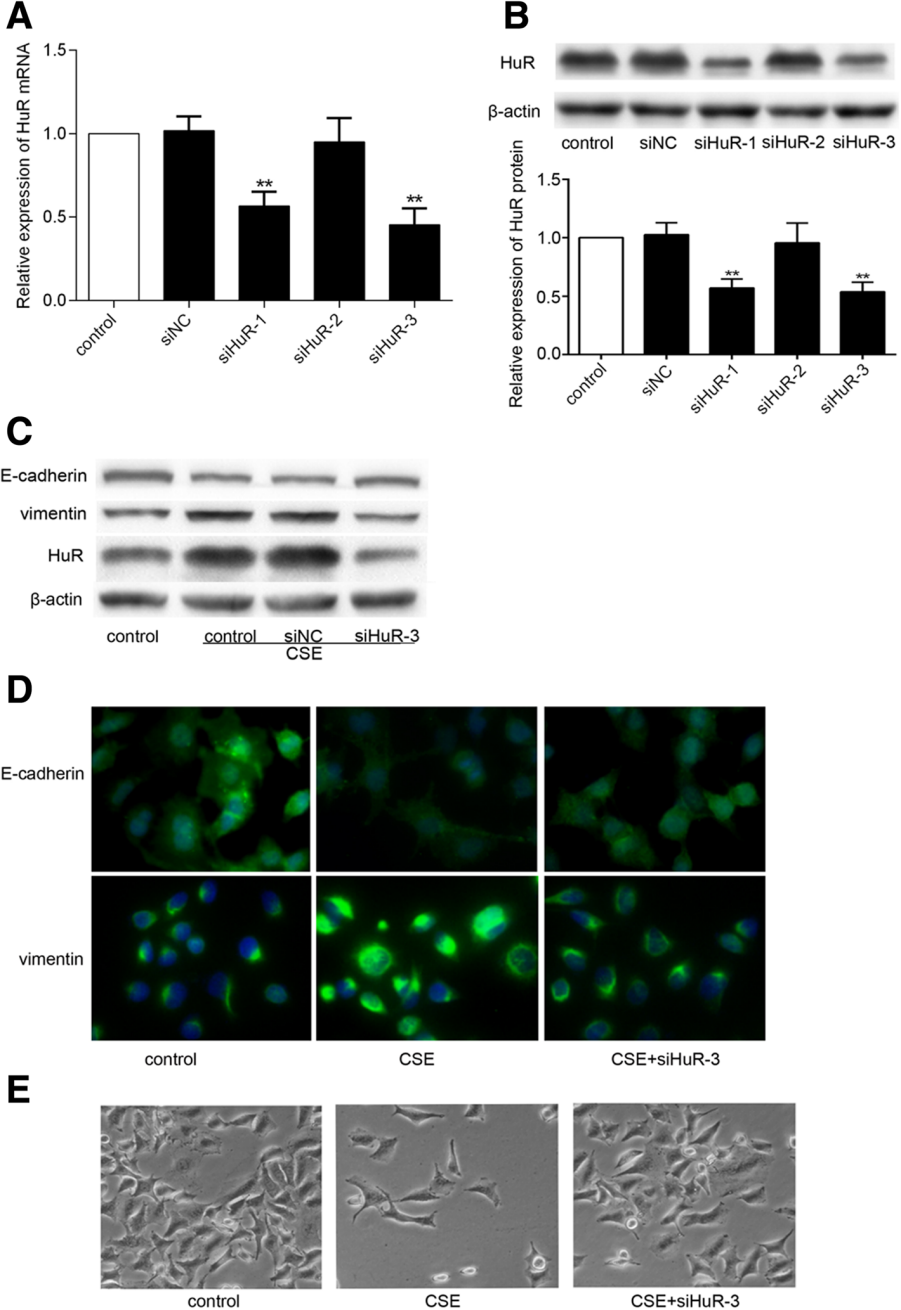
HuR was necessary for CSE-induced EMT in BEAS-2B cells. HuR mRNA (**a**) and protein (**b**) expression levels were detected using real-time PCR and Western blot analysis, respectively, with three HuR-siRNA sequences and a negative control siRNA at 48 h after transfection. Each value is presented as the mean ± SD from three independent experiments. **P < 0.01 compared with cells transfected with siNC. **c**, **d** and **e**) BEAS-2B cells were treated with 3% CSE for 48 h in the presence or absence of HuR gene silencing. **c** Western blot was used to detect the expression of E-cadherin, vimentin, and HuR. **d** Immunofluorescence analysis of the expression of E-cadherin and vimentin using an inversion fluorescence microscope. **e** Cell images were obtained using phase-contrast microscopy

To test whether HuR was required for CSE-induced EMT of BEAS-2B cells, we performed targeted knockdown of HuR using siRNA gene silencing. BEAS-2B cells were transiently transfected with either one of three HuR siRNAs (siHuR-1, siHuR-2 and siHuR-3) or a negative control siRNA (siNC) and harvested for analysis 48 h after transfection. Real-time PCR (Fig. 3a) and Western blot (Fig. 3b) showed that siHuR-1 and siHuR-3 clearly suppressed HuR expression. We choose siHuR-3 (the most effective siRNA) for all subsequent experiments.

Western blot (Fig. 3c) and immunofluorescence analysis (Fig. 3d) showed that after CSE exposure, the levels of E-cadherin (an epithelial marker) were decreased, and the levels of vimentin (a mesenchymal marker) were increased. In contrast, the decrease of E-cadherin expression and the increase of vimentin expression were abolished in cells treated with siHuR-3 and CSE. Moreover, the cell morphological changes were also observed. As shown in Fig. 3e, after a 48 h treatment with 3% CSE, BEAS-2B cells exhibited fewer cell–cell contacts and had a spindle-like shape compared to untreated cells. After transfected with siHuR-3, the cells restored their epithelial morphology. These data suggested that HuR was responsible for CSE-induced EMT of BEAS-2B cells.

### HuR mediated CSE-induced EMT by stabilizing ZEB-1 mRNA in BEAS-2B cells

We proposed that some target mRNAs of HuR may be responsible for the CSE-induced mesenchymal transformation observed in BEAS-2B cells. Recent studies have indicated that zinc finger E-box binding homeobox 1 (ZEB-1), a well-studied transcription factor involved in carcinogenesis, plays a pivotal role in promoting EMT and could be regulated by RNA-binding proteins. We hypothesized that HuR promoted CSE-induced EMT in BEAS-2B cells by stabilizing ZEB-1 mRNA.

**Fig. 4:**
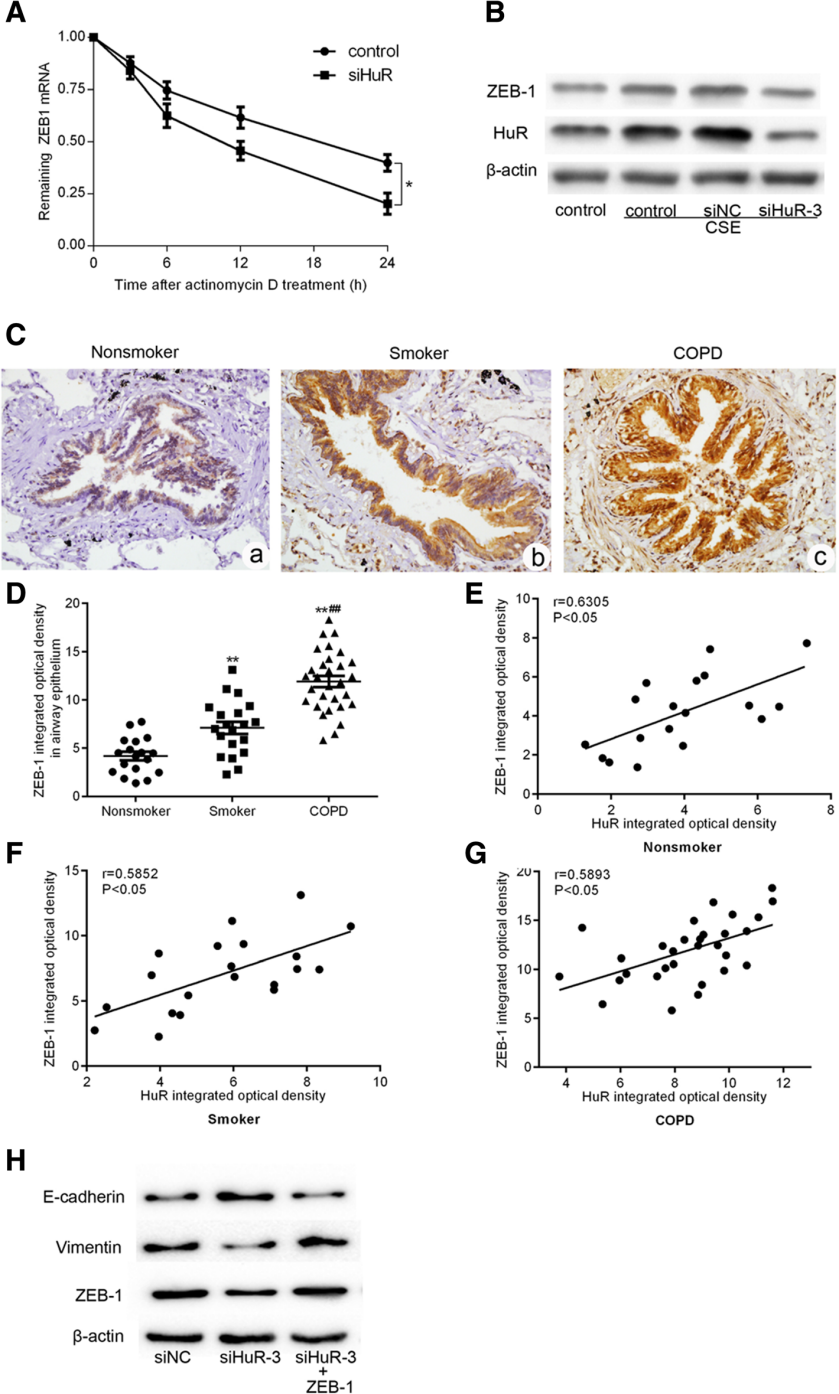
ZEB-1 was required for HuR-mediated EMT in BEAS-2B cells. (A)*P < 0.05 based on a Student’s t-test. (B) Western blot was used to detect ZEB-1 and HuR expression. BEAS-2B cells were treated with 3% CSE for 48 h in the presence or absence of HuR gene silencing. ZEB-1 expression and its correlation with HuR expression in the airway epithelium. Immunohistochemical assessment of ZEB-1 expression in non-smoking subjects (*n* = 18), smokers without COPD (*n* = 20), and smokers with COPD (*n* = 30). (C) Representative ZEB-1 immunostaining (brown staining) in the airways of a nonsmoker (a), a smoker without COPD (b), and a smoker with COPD (c). (D) Quantification of ZEB-1 protein levels in the airway epithelium using the integrated optical mean density. The expression of ZEB-1 was correlated with the HuR expression in the airway epithelium of nonsmokers (E), smokers without COPD (F), and smokers with COPD (G) by Spearman’s correlation test. Western blot was used to detect the expression of ZEB-1, E-cadherin and vimentin (H).The values are given as the mean ± s.e.m. **P < 0.01 compared with nonsmokers and ##*P* < 0.01 compared with smokers without COPD

Upon detection of the stability of ZEB-1 mRNA in cells transfected with siHuR, the half-life of ZEB-1 mRNA was significantly shortened in the HuR-silenced group versus that of the control group (Fig. 4A), suggesting that HuR might enhance ZEB-1 mRNA stability. More evidence was found at the protein level as well. Western blot analysis showed that ZEB-1 was up-regulated by CSE treatment but down-regulated after silencing HuR expression in CSE-treated cells (Fig. 4B). Immunohistochemistry analysis showed that ZEB-1 levels were increased in smokers with COPD compared to those in smokers and nonsmokers without COPD, which was very similar with the expression pattern of HuR(Fig. 4C and D). Statistical analysis showed that ZEB-1expression was positively correlated with HuR level in airway epithelium (Fig. 4E, F and G). Functionally, overexpression of ZEB-1 in HuR-silenced BEAS-2B cells resulted in an obvious decrease of E-cadherin expression and the increase of vimentin expression (Fig. 4H). These data indicated that ZEB-1 mRNA stabilization regulated by HuR was required for CSE-induced EMT in BEAS-2B cells.

## Discussion

COPD is accompanied by inflammation and tissue remodeling [15]. Tissue remodeling in COPD is characterized by emphysema and small airway remodeling with peribronchiolar fibrosis [16]. EMT is a process by which epithelial cells gradually lose their cell polarity and cell-cell adhesions and acquire migratory and invasive properties similar to a mesenchymal phenotype [17]. It has been reported that EMT can cause airway remodeling/fibrosis in COPD, as increased expression of EMT markers is accompanied by reticular basement membrane fragmentation and reduced expression of epithelial junction molecules in the airways of smokers [18, 19]. Mahmood MQ et al. found that there was increased expression of EMT-related markers(EGFR, vimentin, S100A4 and fragmentation) in chronic airflow limitation small airways compared to controls. The result indicated that EMT may be relevant to the key pathologies of chronic obstructive pulmonary disease, small airway fibrosis, and airway cancers [18]. Recent investigations found that nicotine and tobacco smoke could induce EMT in BECs via the Wnt3α/β-catenin/TGF-β pathway [20]. It has been reported that the EMT biomarkers in airway epithelium of COPD patients varied in varying degrees after the treatment of inhaled fluticasone propionate (fluticasone; 500 μg twice daily for 6 months). The result provided strong suggestive support for an anti-EMT effect of ICS in COPD airways [21]. However, the mechanisms leading to EMT in the airways of patients with COPD remain largely unknown.

The RNA-binding protein HuR is one of the best-studied regulators of cytoplasmic mRNAs fate. Through post-transcriptional influence on target mRNAs, HuR can adjust the cellular response to inflammatory, proliferative, differentiation, senescence, apoptotic, stress and immune stimuli [14]. We have previously shown that altered expression and activity of HuR participated in PDGF-induced human airway smooth muscle cell proliferation and expression of cyclin D1 [13]. Additionally, we demonstrated that an HuR/TGF-β1 feedback circuit was established to regulate airway remodeling in vivo and in vitro and that targeting this feedback loop has considerable potential for treating asthma [22]. Thus, these phenomena indicate that HuR might be a significant factor which is responsible for airway remodeling in asthma. But, to date, there is no report on whether HuR is implicated in COPD pathogenesis, especially in airway fibrosis in COPD.

In this study, we demonstrate for the first time that HuR expression altered in airway epithelium of COPD subjects. In smokers without COPD, the HuR expression levels were higher than those in nonsmokers. Moreover, the expression of HuR in smokers with COPD was obviously higher than that in the other two groups. This phenomenon indicates that HuR could play a significant role in the pathogenesis of COPD. The pathological process is associated with exposure to cigarette smoke. Although Hudy et al. [23] reported that CSE does not induce dysregulation of the RBPs AUF1 and HuR in primary human bronchial epithelial cells, the altered expression of HuR was validated in BEAS-2B cells after treatment with CSE in our research. Aside from enhancing HuR expression, our results also incicate that CSE could promote the translocation of HuR from nucleus to the cytoplasm. As the most prominent RNA-binding protein, HuR is predominantly localized in the nucleus when the cell is in a quiescent state. Once activated, HuR could rapidly translocate from nuclear to cytoplasm, where it will exert its RNA-binding activities. The increased cytoplasmatic HuR levels might indicate that CSE could enhance the activities of HuR. Similar results were also demonstrated in Michela Zago’s study [24].

Another major finding of our present study was that lowering HuR expression using RNA-interference could effectively decrease CSE-induced EMT of BEAS-2B cells. After CSE exposure, BEAS-2B cells showed lower expression of E-cadherin and higher expression of vimentin, as well as exhibiting a mesenchymal phenotype. Nevertheless, the aforementioned changes were significantly reversed by HuR silencing, which indicates that HuR is required for CSE-induced EMT. Such results are in accordance with the emerging role of HuR in regulating the EMT process shown as in Wan Q’s study [9].

Several transcription factors, such as Snail, Slug, ZEB-1, ZEB-2, Twist and β-catenin, have been identified as key regulators of EMT and have been extensively reported [25–29]. In Mahmood MQ’s study [25], β-catenin and Snail 1 expression was generally high in all subjects throughout the airway wall with marked cytoplasmic to nuclear shift in COPD. Moreover, Twist expression was generalised in the epithelium in normal but become more basal and nuclear with smoking. In our effort to elucidate the mechanism how HuR modulates EMT in BEAS-2B cells, we identified ZEB-1 as an effective mediator of these HuR-induced phenomena. It is widely accepted that ZEB-1 is involved in cancer invasion in different tumors, including breast cancer [26], renal cell carcinoma [27] and esophageal squamous cancer [28]. It is known that HuR silencing decreases ZEB-1 protein expression suggesting that HuR is involved in modulation of this gene. But, the mechanistic connection between HuR and ZEB-1 in CSE-induced EMT and in COPD was previously unknown. In this study, we showed that modulation of HuR expression altered the half-life of ZEB-1 mRNA and post-transcriptionally controlled ZEB-1 expression. Functionally, ZEB-1 is required for HuR-mediated EMT in BEAS-2B cells. Our study reveals a mechanism by which HuR promotes CSE-induced EMT through increasing ZEB-1 expression.

The previous reports showed that EMT could occur both in large airways [30] and small airways of COPD patients. In this study, we focus on the EMT process in small airways (2.5 mm internal diameter). Actually, we didn’t observe the HuR and ZEB-1 expression in large airways. We think that whether HuR and ZEB-1 play the same role in large airways will be the next step of our research.

## Conclusion

In conclusion, our findings provide compelling evidence for the role and the mechanism of HuR in the development and progression of EMT in an aggressive phenotype in COPD. Considering that EMT is controlled by a series of transcriptional and post-transcriptional regulators, the gene regulatory network will be fundamental to understanding relative disease progression. A better comprehension of the mechanisms underlying the bronchial epithelial cell plasticity conferred by EMT programming might facilitate the design of therapeutic interventions aimed at targeting selected signaling pathways to prevent COPD initiation and progression.

## References

[1] Burney P, Kato B, Janson C, Mannino D, Studnicka M, Tan W, Bateman E, Koçabas A, Vollmer WM, Gislason T, Marks G, Koul PA, Gnatiuc L, Buist S (2014). Burden of obstructive lung disease (BOLD) study. Chronic obstructive pulmonary disease mortality and prevalence: the associations with smoking and poverty--a BOLD analysis. Thorax.

[2] Vogelmeier CF, Criner GJ, Martinez FJ, Anzueto A, Barnes PJ, Bourbeau J, Celli BR, Chen R, Decramer M, Fabbri LM, Frith P, Halpin DM, López Varela MV, Nishimura M, Roche N, Rodriguez-Roisin R, Sin DD, Singh D, Stockley R, Vestbo J, Wedzicha JA, Agustí A. Global strategy for the diagnosis, management, and prevention of chronic obstructive lung disease 2017 report. GOLD executive summary. Am J Respir Crit Care Med 2017 *Mar 1;*195*(*5*):*557*–*582*.* 10.1164/rccm.201701-0218PP 28128970

[3] Tam A, Sin DD (2012). Pathobiologic mechanisms of chronic obstructive pulmonary disease. Med Clin North Am.

[4] Calverley PM, Anderson JA, Celli B, Ferguson GT, Jenkins C, Jones PW, Yates JC, Vestbo J (2007). TORCH investigators. Salmeterol and fluticasone propionate and survival in chronic obstructive pulmonary disease. N Engl J Med.

[5] Young RP, Whittington CF, Hopkins RJ, Hay BA, Epton MJ, Black PN, Gamble GD (2010). Chromosome 4q31 locus in COPD is also associated with lung cancer. Eur Respir J.

[6] Nowrin K, Sohal SS, Peterson G, Patel R, Walters EH (2014). Epithelial-mesenchymal transition as a fundamental underlying pathogenic process in COPD airways: fibrosis, remodeling and cancer. Expert Rev Respir Med.

[7] Kelley DR, Hendrickson DG, Tenen D, Rinn JL (2014). Transposable elements modulate human RNA abundance and splicing via specific RNA-protein interactions. Genome Biol.

[8] Wang J, Guo Y, Chu H, Guan Y, Bi J, Wang B (2013). Multiple functions of the RNA-binding protein HuR in cancer progression, treatment responses and prognosis. Int J Mol Sci.

[9] Yu C, Xin W, Zhen J, Liu Y, Javed A, Wang R, Wan Q (2015). Human antigen R mediated post-transcriptional regulation of epithelial-mesenchymal transition related genes in diabetic nephropathy. Journal of diabetes.

[10] Wang Q, Wang Y, Zhang Y, Zhang Y, Xiao W (2013). The role of uPAR in epithelial-mesenchymal transition in small airway epithelium of patients with chronic obstructive pulmonary disease. Respir Res.

[11] Aoshiba K, Nagai A, Konno K (1994). Nicotine prevents a reduction in neutrophil filterability induced by cigarette smoke exposure. Am J Respir Crit Care Med.

[12] Wang LN, Chen WW, Zhang J, Li CY, Liu CY, Xue J, Zhang PJ, Jiang AL (2013). The miRNA let-7a1 inhibits the expression of insulin-like growth factor 1 receptor (IGF1R) in prostate cancer PC-3 cells. Asian J Androl.

[13] Zhang P, Cao M, Liu Y, Lv Z, Yang Q, Lin X, Li H, Wan Q (2012). PDGF-induced airway smooth muscle proliferation is associated with human antigen R activation and could be weakened by AMPK activation. Mol Biol Rep.

[14] Srikantan S, Gorospe M (2012). HuR function in disease. Frontiers in bioscience (Landmark edition).

[15] de-Torres JP, Sanchez-Salcedo P, Bastarrika G, Alcaide AB, Pio R, Pajares MJ, Campo A, Berto J, Montuenga L, Del Mar Ocon M, Monente C, Celli BR, Zulueta JJ. Telomere length, COPD and emphysema as risk factors for lung cancer. Eur Respir J. 2017:49(1). 10.1183/13993003.01521-2016 28049172

[16] Zhou-Suckow Z, Duerr J, Hagner M, Agrawal R, Mall MA. Airway mucus, inflammation and remodeling: emerging links in the pathogenesis of chronic lung diseases. Cell Tissue Res. 2017; 10.1007/s00441-016-2562-z 28108847

[17] Sohal SS, Walters EH (2013). Epithelial mesenchymal transition (EMT) in small airways of COPD patients. Thorax.

[18] Tian B, Zhao Y, Sun H, Zhang Y, Yang J, Brasier AR (2016). BRD4 mediates NF-kappaB-dependent epithelial-mesenchymal transition and pulmonary fibrosis via transcriptional elongation. American journal of physiology Lung cellular and molecular physiology.

[19] Mahmood MQ, Sohal SS, Shukla SD, Ward C, Hardikar A, Noor WD, Muller HK, Knight DA, Walters EH (2015). Epithelial mesenchymal transition in smokers: large versus small airways and relation to airflow obstruction. International journal of chronic obstructive pulmonary disease.

[20] Zou W, Zou Y, Zhao Z, Li B, Ran P (2013). Nicotine-induced epithelial-mesenchymal transition via Wnt/beta-catenin signaling in human airway epithelial cells. American journal of physiology Lung cellular and molecular physiology.

[21] Sohal SS, Soltani A, Reid D, Ward C, Wills KE, Muller HK, Walters EH (2014). A randomized controlled trial of inhaled corticosteroids (ICS) on markers of epithelial-mesenchymal transition (EMT) in large airway samples in COPD: an exploratory proof of concept study. Int J Chron Obstruct Pulmon Dis.

[22] Wang N, Yan D, Liu Y, Liu Y, Gu X, Sun J, Long F, Jiang S (2016). A HuR/TGF-beta1 feedback circuit regulates airway remodeling in airway smooth muscle cells. Respir Res.

[23] Hudy MH, Proud D (2013). Cigarette smoke enhances human rhinovirus-induced CXCL8 production via HuR-mediated mRNA stabilization in human airway epithelial cells. Respir Res.

[24] Zago M, Sheridan JA, Nair P, Rico de Souza A, Gallouzi IE, Rousseau S, Di Marco S, Hamid Q, Eidelman DH, Baglole CJ (2013). Aryl hydrocarbon receptor-dependent retention of nuclear HuR suppresses cigarette smoke-induced cyclooxygenase-2 expression independent of DNA-binding. PLoS One.

[25] Mahmood MQ, Walters EH, Shukla SD, Weston S, Muller HK, Ward C, Sohal SS (2017). β-catenin, twist and snail: transcriptional regulation of EMT in smokers and COPD, and relation to airflow obstruction. Sci Rep.

[26] Preca BT, Bajdak K, Mock K, Lehmann W, Sundararajan V, Bronsert P, Matzge-Ogi A, Orian-Rousseau V, Brabletz S, Brabletz T, Maurer J, Stemmler MP (2017). A novel ZEB1/HAS2 positive feedback loop promotes EMT in breast cancer. Oncotarget.

[27] Sun KH, Sun GH, Wu YC, Ko BJ, Hsu HT, Wu ST (2016). TNF-alpha augments CXCR2 and CXCR3 to promote progression of renal cell carcinoma. J Cell Mol Med.

[28] Ohashi S, Natsuizaka M, Naganuma S, Kagawa S, Kimura S, Itoh H, Kalman RA, Nakagawa M, Darling DS, Basu D, Gimotty PA, Klein-Szanto AJ, Diehl JA, Herlyn M, Nakagawa H (2011). A NOTCH3-mediated squamous cell differentiation program limits expansion of EMT-competent cells that express the ZEB transcription factors. Cancer Res.

[29] Mahmood MQ, Reid D, Ward C, Muller HK, Knight DA, Sohal SS, Walters EH (2017). Transforming growth factor (TGF) β1 and Smad signalling pathways: a likely key to EMT-associated COPD pathogenesis. Respirology.

[30] Sohal SS, Reid D, Soltani A, Ward C, Weston S, Muller HK, Wood-Baker R, Walters EH (2010). Reticular basement membrane fragmentation and potential epithelial mesenchymal transition is exaggerated in the airways of smokers with chronic obstructive pulmonary disease. Respirology.

